# Efficacy of Conservative Therapy in Overhead Athletes with Glenohumeral Internal Rotation Deficit: A Systematic Review and Meta-Analysis

**DOI:** 10.3390/jcm12010004

**Published:** 2022-12-20

**Authors:** Sandra Jiménez-del-Barrio, Luis Ceballos-Laita, Almudena Lorenzo-Muñoz, María Teresa Mingo-Gómez, Manuel Rebollo-Salas, José Jesús Jiménez-Rejano

**Affiliations:** 1 Clinical Research Group in Health Sciences, Department of Surgery, Ophthalmology and Physiotherapy, Faculty of Health Sciences, University of Valladolid, 42004 Soria, Spain; 2 Department of Physiatry and Nursery, Faculty of Health Sciences, University of Zaragoza, 50009 Zaragoza, Spain; 3 Department of Health and Sports, Pablo Olavide University, 41013 Sevilla, Spain; 4 Department of Physiotherapy, University of Sevilla, 41004 Sevilla, Spain

**Keywords:** overhead athletes, shoulder, joint hypomobility, systematic review, meta-analysis

## Abstract

Background: To evaluate the effectiveness of conservative therapy in range of movement (ROM), strength, pain, subacromial space and physical function, in overhead athletes with glenohumeral internal rotation deficit (GIRD). Methods: A systematic review and meta-analysis was designed, and the protocol was registered in PROSPERO (CRD42021281559). The databases searched were: PubMed, Cochrane Central Register of Controlled Trials, Physiotherapy Evidence Database, Web of Science and SCOPUS. Randomized controlled trials (RCTs) involving conservative therapy applied in overhead athletes with GIRD were included. Two independent assessors evaluated the quality of the studies with the PEDro scale, and with the Cochrane Risk-of-Bias tool. The overall quality of the evidence was assessed using GRADE. Data on outcomes of interest were extracted by a researcher using RevMan 5.4 software. Estimates were presented as standardized mean differences (SMD) with 95% confidence intervals (CIs). Results: A total of eleven studies involving 514 overhead athletes were included in the systematic review; of these 8 were included in the meta-analysis. The methodological quality of the included RCTs ranged from high to low. Conservative therapy showed significant improvements in internal rotation, adduction, physical function and subacromial space. Conclusions: Conservative therapy based on stretch, passive joint and muscular mobilizations can be useful to improve the internal rotation and adduction ROM, subacromial space, and physical function of the shoulder in overhead athletes with glenohumeral internal rotation deficit.

## 1. Introduction

Overhead athletes are characterized as having developed specific asymmetrical adaptations to their dominant upper limbs. That fact affects their ROM, changing the distribution of the muscular load [1]. Sometimes, non-physiological loads lead to stress musculoskeletal structures, and they consequently develop pain and overuse injuries [2]. Overhead athletes present with a high prevalence of shoulder injury. Lifetime prevalence of shoulder pain was determined for elite athletes at 41.6% [3,4].

Several cinematic risk factors have been investigated for shoulder overuse injuries. The GIRD has been described as the primary risk factor for overuse injuries. Recent studies have demonstrated that overhead athletes with GIRD present a higher risk of suffering injury than those without a restricted ROM [5]. In addition, external rotation gain (ERG) or loss of total ROM have been considered as a potential risk factors in this dysfunction [5,6,7,8]. These risk factors have shown to be more frequent in sports like baseball, volleyball, and handball.

Clinical guidelines and RCTs have proposed different conservative therapies to improve this condition [9,10,11,12]. Several authors have concluded that non-pharmacological conservative therapies, such as manual therapy and therapeutic exercise, are effective in reducing pain, improving shoulder ROM, and physical function in patients with shoulder dysfunctions [11,13,14,15].

Steuri et al. and Dolder et al. proposed exercise, manual therapy, tape, electrotherapy, or soft tissue mobilizations as feasibility tools to manage shoulder restriction ROM in adults with shoulder pain [16,17]. Recent clinical trials have been developed to demonstrate the effects of conservative therapy on symptoms, shoulder ROM and physical function in overhead athletes, mostly focusing on achieving greater joint mobility with manual therapy, stretching, and self-stretching or strengthening exercises [18,19,20,21]. However, the effects of these conservative therapies on the management of the main clinical signs in overhead athletes with GIRD remain unclear. Thus, a systematic review with meta-analysis comprising the effects of non-pharmacological conservative therapies in overhead athletes with GIRD is needed.

The current systematic review with meta-analysis investigated the effectiveness of conservative therapy in ROM, strength, pain and physical function in overhead athletes with GIRD.

## 2. Materials and Methods

This systematic review followed the Preferred Reporting Items for Systematic Reviews and Meta-analysis (PRISMA) statement and Cochrane recommendations [22]. The protocol was registered in PROSPERO (CRD42021281559).

### 2.1. Data Sources and Searches

Search strategies were conducted on PubMed, Cochrane Central Register of Controlled Trials, PEDro, Web of Science (WoS), SCOPUS databases up to July 2022. Our search terms included RCTs involving overhead athletes with GIRD and/or shoulder pain during physical practice that compared a conservative therapy against a control, sham, or other conservative therapy. There was no restriction on publication year or language. The search strategy is provided in Appendix A.

### 2.2. Study Selection

The included studies met the PICOs criteria: (1) overhead athletes with GIRD; (2) interventions studied were conservative therapies; (3) comparison was control, sham, or other conservative therapy; (4) outcomes consisted of shoulder internal rotation ROM, external rotation ROM, internal and external shoulder strength, pain and/or physical function; and (5) studies were RCTs.

Studies were excluded if they: (1) were trials conducted with animals, cadavers, or simulators; (2) included participants suffering traumatic injuries, or after surgical interventions; (3) and did not report outcomes of interest.

After searches were retrieved, references were exported to Mendeley desktop, and duplicates were removed. Two reviewers independently (S.J.-d.-B. and L.C.-L.) assessed the title and abstract of each reference to determine potential eligibility. Potential full texts were assessed by the same independent reviewers. Any discrepancies between the two reviewers were resolved by a third author (J.J.J.-R.). Two authors were contacted by e-mail to clarify eligibility criteria.

### 2.3. Data Extraction

We analyzed the data using a qualitative synthesis and, whenever possible, using a quantitative synthesis (meta-analysis).

The following data were extracted by two independent assessors (S.J.-d.-B. and L.C.-L.) from included articles using standardized forms: citation details, study design, purpose, description of participants, interventions and outcome measures and results. Any disagreements were resolved by a third assessor (J.J.J.-R.). Kappa coefficient was calculated to assess the agreement.

### 2.4. Methodological Quality Appraisal

Quality of the studies was assessed by two assessors using the PEDro scale and the Cochrane Risk of Bias tool. Both tools have shown to be reliable for evaluating the quality of studies and assessing the risk of bias.

### 2.5. Data Synthesis and Analysis

The quantitative synthesis of the results was carried out according to the outcomes considered: shoulder internal, external rotation and adduction ROM; pain; physical function; muscular strength; and subacromial space. When studies used the same tools to assess the same outcome, the authors utilized the continuous data method. When different tools were used, the authors utilized inverse variance methods.

Eight different meta-analyses were performed for results of internal rotation and external rotation, adduction ROM, internal and external rotation shoulder strength, pain intensity, physical function, and subacromial space. To perform the meta-analysis, means post intervention scores and standard deviations (SDs) were used.

SMD and 95% CI were calculated based on the post-intervention means and SDs. Subgroup analyses of studies were performed to compare different types of intervention groups with control, sham, or other conservative therapies. Significance was set at a *p* value < 0.05. Statistical heterogeneity was assessed using the inconsistency measure (I^2^). Fixed or random effects models were used according to the degree of heterogeneity, which was assessed using the I^2^ coefficient. If I^2^ was superior to 50%, or if the *p* value inferior or equal than 0.05 indicated heterogeneity, then random effects models were used; when I^2^ was less than 50% or the *p* value was superior to 0.05, fixed effect models were used.

Review Manager with the RevMan version 5.4 software was used to perform meta-analysis on the outcomes of interest data. and Results were presented graphically.

### 2.6. Overall Quality of the Evidence

GRADE Evidence Profiles were constructed, and Evidence Tables were produced to determine the rating of the evidence. The ratings were defined as follows. High certainty ⊕⊕⊕⊕: very confident that the true effect is close to the effect estimate. Moderate certainty ⊕⊕⊕◯: moderately confident in the effect estimate; the true effect is likely to be close to the effect estimate but may differ substantially. Low certainty ⊕⊕◯◯: limited confidence in the effect estimate; the true effect may differ substantially from the effect estimate. Very low certainty ⊕◯◯◯: confidence in the estimate of the effect is very low. GRADE assesses quality according to the following domains: study design, risk of bias, inconsistency, indirect evidence, imprecision, and other factors.

## 3. Results

**Figure 1:**
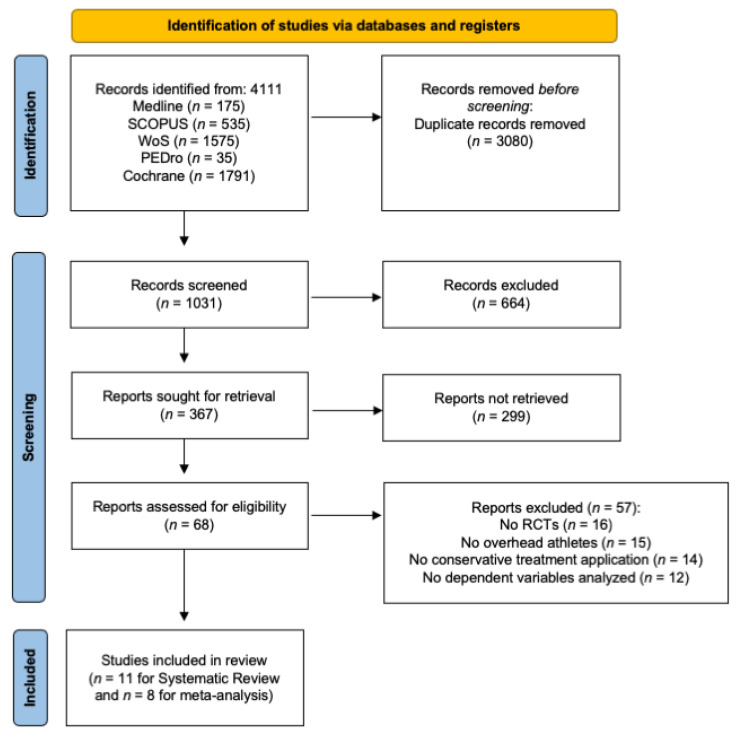
Flowchart diagram of study selection process.

Eleven studies were included in the qualitative synthesis, and 8 of those in the quantitative synthesis. The description of the selection process can be found in the PRISMA flow diagram (Figure 1). The agreement between reviewers was calculated by kappa with a value of 0.9.

### 3.1. Characteristics of the Eligible Studies

We included a total of 11 RCTs comprising 514 overhead athletes with GIRD. The sample size ranged from 30 to 88 patients. The sports involved were mostly handball, baseball, and volleyball.

All studies considered the GIRD as a diagnostic criterion. Two studies included athletes with a GIRD > 15° [23,24], four studies with a GIRD < 15° [20,21,25,26], and two studies included athletes with a 15° deficit in the total arc of motion (15° deficit in internal rotation or/and 15° deficit in horizontal adduction) [27,28]. Another study considered a reduction greater than 10% for the inclusion [29]. Three studies considered the presence of shoulder pain as well [26,30,31]. The sociodemographic and clinical characteristics of the participants in each study are shown in Table 1.

The intervention in each trial consisted of different approaches. Eight studies applied self-stretching or passive stretching [20,23,24,25,26,27,28,29]; six studies used manual therapy, frequently based on anteroposterior mobilization [24,26,27,30,31,32]; two studies applied exercise therapy [23,31] and one study used a kinesiotaping application [29]. Three studies mixed conservative therapy (self-stretching plus manual therapy [24,27] and exercise therapy associated with manual therapy [26]).

Table 1 shows the studies based on conservative therapies focused on passive or active interventions compared to control, sham, or other conservative therapy intervention.

The number of sessions involved varied for different studies. The studies in which exercise therapy was applied were performed for 8 weeks [23,31]; when stretching was used, the duration was between 4 to 8 weeks [20,25,28]. The rest of the conservative therapies’ interventions varied in the duration of the studies. Instrumental therapies were applied during one session [21,30], three sessions [26] or up to 2 weeks [24].

### 3.2. Outcome Measures

We performed 8 different meta-analyses; one for each dependent variable analyzed in the study. The joint mobility was considered, including internal rotation, external rotation, and adduction ROM. These variables were evaluated using an inclinometer in six studies [20,21,23,24,25,27], a goniometer in three studies [28,29,30], or photogrammetry in one study [26]. Muscular strength was evaluated using a handheld dynamometer. Four studies assessed internal and external rotation strength [21,29,30,31]. In one study, strength was evaluated by electromyography and isokinetic dynamometer [31]. Pain variable was considered and was measured by numeric pain rate scale (NPRS) in 4 studies [20,21,28,30]. To evaluate the subacromial space, we relied on two studies using ultrasonography [27,29]. Physical function was analyzed using NPRS in one study [28] and Disability of the arm, shoulder and hand (DASH) questionnaire in another study [30].

### 3.3. Methodological Quality of Included Studies

**Figure 2:**
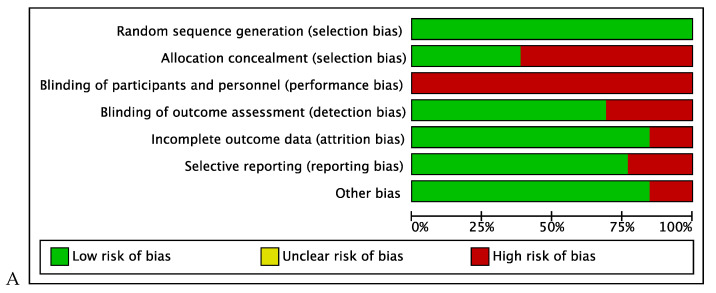

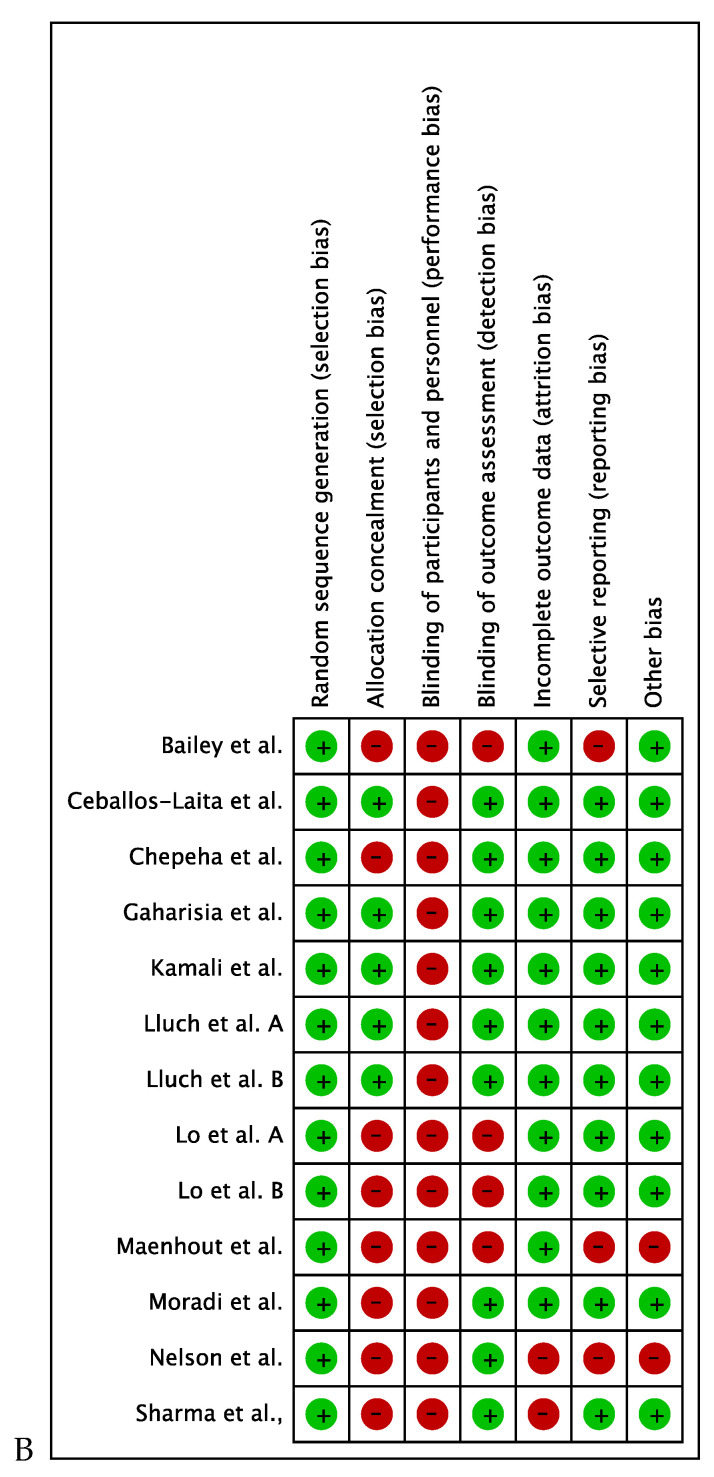
Risk of bias. + = Low risk of bias, - = High risk of bias. (**A**) Presents the Risk of bias of all the studies according to the Cochrane Tool Risk of Bias items. (**B**) Shows the Risk of bias specifically of each study according to the Cochrane Tool Risk of Bias items.

All the RCTs included in this review described a low risk of selection bias and reporting. Most of studies used random allocation, with the groups similar at baseline. No studies blinded the participants or therapist, which is expected, as the studies involved physiotherapy. Eight studies blinded the outcome assessment [20,21,23,24,26,28,30,31]. The mean score of the trials was 6.54 on the PEDro scale (ranged from 4 to 9) (Table 1). The Cochrane risk-of-bias tool results are shown in Figure 2.

### 3.4. Synthesis of Results

#### 3.4.1. Internal Rotation ROM

**Figure 3:**
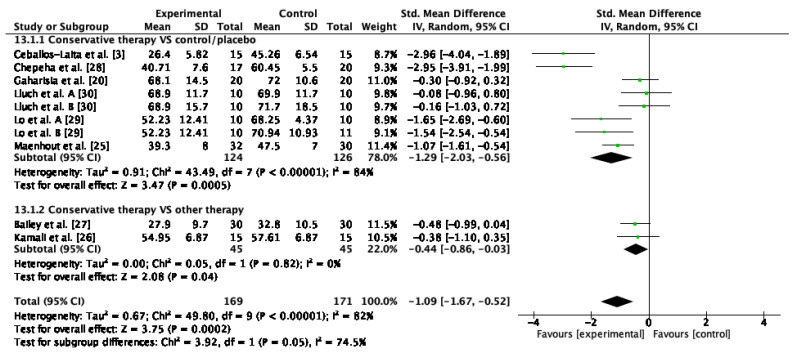
Forest plot of internal rotation ROM for conservative therapy versus control/sham and combination of conservative therapies versus conventional therapy in isolation. Lluch et al. A. compared manual therapy versus control intervention. Lluch et al. B compared the manual contact versus control intervention. Lo et al. A compared KT versus control intervention. Lo et al. B compared stretching versus control intervention.

The internal rotation ROM was measured in 10 studies [20,21,23,24,25,26,27,28,29,30]. Concerning qualitative analysis, positive results were observed in nine studies after conservative therapy application [20,21,23,25,26,27,28,29,30] (Table 1). Eight studies included in the quantitative synthesis and meta-analysis showed a significant improvement in internal rotation ROM after conservative treatment [20,21,25,26,27,28,29,30] in comparison to control or sham treatment (SMD = −1.29; 95% CI: −2.03,−0.56; I^2^: 84%; *p* < 0.01). The improvement in internal rotation ROM from passive mobilization plus stretching compared to stretching in isolation was significant (SMD = −0.44; 95% CI: −0.86, −0.03; I^2^: 0%; *p* = 0.04) (Figure 3). The global result achieved significant changes between groups (SMD = −1.09; 95% CI: −1.67, −0.52; I^2^: 82%; *p* < 0.01).

#### 3.4.2. External Rotation ROM

**Figure 4:**
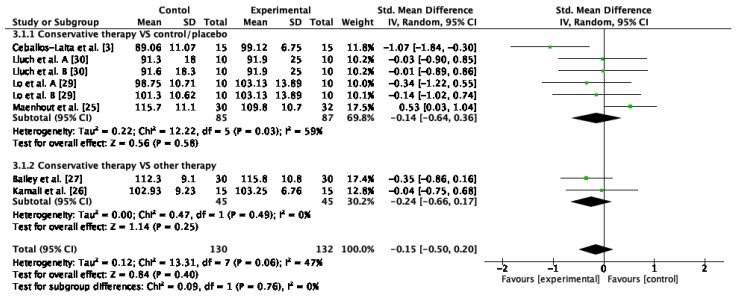
Forest plot of external rotation ROM for conservative therapy versus control/sham and combination of conservative therapies versus conventional therapy in isolation. Lluch et al. A. compared manual therapy versus control intervention. Lluch et al. B compared the manual contact versus control intervention. Lo et al. A compared KT versus control intervention. Lo et al. B compared stretching versus control intervention.

The external rotation ROM was measured in six studies [21,25,26,27,29,30], which were included in the quantitative synthesis and meta-analysis for the external rotation ROM. The results of the meta-analysis indicated that there were no improvements after conservative therapy compared to control or sham (SMD = −0.14; 95% CI: −0.64, 0.36; I^2^: 59%; *p* = 0.58). Conservative therapy based on passive mobilization plus stretching compared to stretching in isolation presented no differences between groups (SMD = −0.24; 95% CI: −0.66, 0.17; I^2^: 0%; *p* = 0.25). The global result achieved significant changes between groups (SMD = −0.15; 95%CI: −0.50, 0.20; I^2^: 47%; *p* = 0.40) (Figure 4).

#### 3.4.3. Adduction ROM

**Figure 5:**
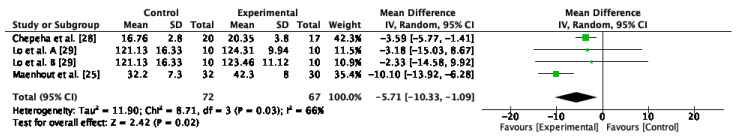
Forest plot of adduction ROM for conservative therapy versus control/sham and conservative therapy versus other conservative therapy. Lo et al. A compared KT versus control intervention. Lo et al. B compared stretching versus control intervention.

The adduction ROM was measured in four studies [25,27,28,29]. The results of the meta-analysis showed significant improvements after conservative therapy compared to control or sham (SMD = −0.77; 95% CI: −0.19, −1.34; I^2^: 59%; *p* < 0.01; MD= −5.71; 95% CI: −1.09, −10.33; I^2^: 66%; *p* = 0.02;) (Figure 5).

#### 3.4.4. Internal Rotation Strength

**Figure 6:**
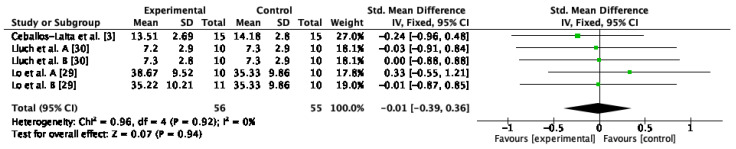
Forest plot of internal rotation strength for conservative therapy versus control/sham. Lluch et al. A. compared manual therapy versus control intervention. Lluch et al. B included the manual contact versus control intervention. Lo et al. A compared KT versus control intervention. Lo et al. B compared stretching versus control intervention.

The internal rotation strength was assessed in four studies [21,29,30,31]. In the qualitative analysis, no differences between groups were observed in the studies after conservative therapy application, as shown in Table 1. The results of the meta-analysis indicated that there were no improvements after conservative therapy compared to control or sham (SMD = −0.01; 95% CI: −0.39, 0.36; I^2^: 0%; *p* = 0.94) (Figure 6).

#### 3.4.5. External Rotation Strength

**Figure 7:**
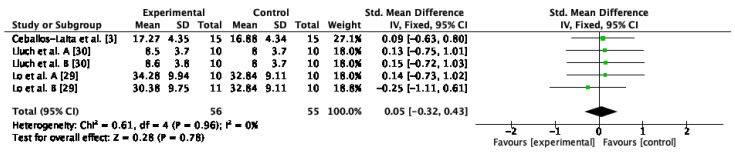
Forest plot of external rotation strength for conservative therapy versus control/sham. Lluch et al. A. compared manual therapy versus control intervention. Lluch et al. B compared themanual contact versus control intervention. Lo et al. A compared KT versus control intervention. Lo et al. B compared stretching versus control intervention.

Four studies assessed external rotation strength [21,29,30,31]. The results of the meta-analysis indicated that there were no improvements after conservative therapy compared to control or sham (SMD = 0.05; 95% CI: −0.32, 0.43; I^2^: 0%; *p* = 0.78) (Figure 7).

#### 3.4.6. Pain Intensity

**Figure 8:**
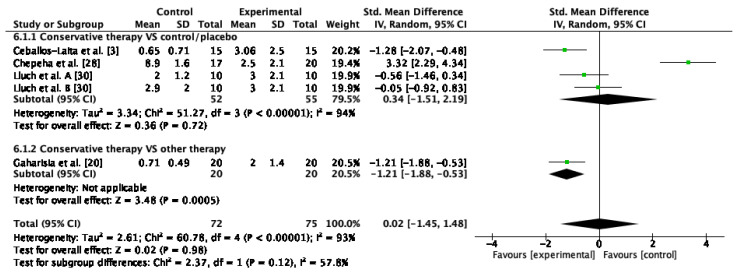
Forest plot of pain intensity for conservative therapy versus control/sham and conservative therapy versus other conservative therapy. Lluch et al. A. compared manual therapy versus control intervention. Lluch et al. B compared the manual contact versus control intervention.

Four studies assessed pain intensity [20,21,28,30]. The results of the meta-analysis indicated that there were no improvements after conservative therapy compared to control or sham or other conservative therapy (SMD = 0.34; 95% CI: −1.51, 2.19; I^2^: 94%; *p* = 0.72). The overall effect showed no significant improvements (SMD = 0.02; 95% CI: −1.45,1.48; I^2^: 93%; *p* = 0.98) (Figure 8).

#### 3.4.7. Physical Function

**Figure 9:**
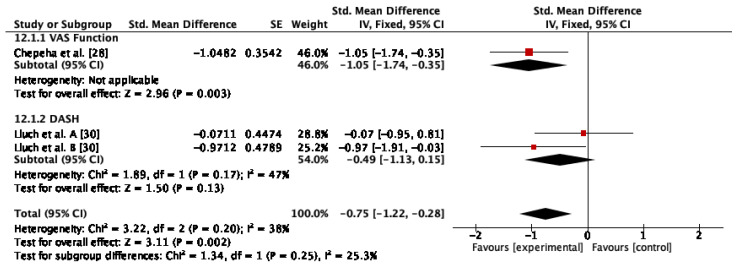
Forest plot of physical function for conservative therapy versus control/sham and conservative therapy versus other conservative therapy. Lluch et al. A. compared manual therapy versus control intervention. Lluch et al. B compared the manual contact versus control intervention.

Two studies assessed physical function [28,30]. The results of the meta-analysis indicated significant changes after conservative therapy compared to control or sham or other conservative therapy (SMD = −0.75; 95% CI: −1.22, −0.28; I^2^: 38%; *p* < 0.01) (Figure 9).

#### 3.4.8. Subacromial Space

**Figure 10:**
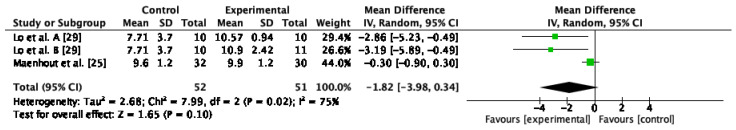
Forest plot of subacromial space for conservative therapy versus control/sham and conservative therapy versus other conservative therapy. Lo et al. A compared KT versus control intervention. Lo et al. B compared stretching versus control intervention.

Two studies assessed subacromial space [27,29]. The results of the meta-analysis indicated significant changes after conservative therapy compared to control or sham or other conservative therapy (SMD = 0.62; 95% CI: −1.18, −0.07; I^2^: 38%; *p* = 0.03; MD = 1.82; 95%CI: −3.98, 0.34; I^2^: 75%; *p* = 0.10) (Figure 10).

### 3.5. GRADE: Quality of Evidence

The overall quality of evidence according to GRADE was rated as high for internal, external rotation, and adduction ROM; moderate for pain, subacromial space, and internal and external rotation strength; and low for physical function. Internal external rotation and adduction ROM were important, while the remaining results of the variables were not important. The overall quality of evidence was moderate by GRADE Criteria (Appendix B).

## 4. Discussion

The present systematic review and meta-analysis shows that conservative therapy appears to be effective for internal rotation and adduction ROM and physical function.

The GRADE classification shows a moderate grade of recommendation for global balance.

The methodological quality was determined to be an average of 6.5 on the PEDro scale. Most of the studies failed in the same items, such as blinding of the therapist who administered the therapy.

The inclusion criterion applied in the studies was internal rotation ROM restriction. Four of the studies included also pain as an inclusion criterion [20,21,28,30]. The inclusion of the internal rotation ROM restriction as eligibility criteria in this review was because it is considered a primary risk factor for the development of shoulder injuries in overhead athletes, even if the athletes do not present shoulder pain [33].

Conservative therapies were applied in the experimental group of all the studies included. The interventions applied were passive and active stretching, manual therapy, soft tissue mobilization and exercise therapy. Two studies used stretching in isolation [25,28]. Two studies combined the stretching with other conservative therapy such as scapula manual mobilization [26] and instrumental soft tissue mobilization [27]. Two studies applied exercise therapy [23,31]. One of the studies applied exercise in combination with manual therapy mobilization [31]. Two studies applied soft tissue mobilization in isolation [21,24], one applied dry needling and the other, fascial manipulation. Finally, one study applied manual therapy mobilizations [30] in isolation, while another used kinesiotaping application [29].

Five of the 11 studies compared the experimental group to a control or sham group. Three of the studies compared the experimental group to a conventional stretching protocol [20,26,27] and one study applied it with mobilization [24]. Two studies applied exercise therapy in the experimental group and used other types of exercise as the comparison [23,31].

The results from this meta-analysis showed that conservative therapy was effective for improving internal rotation and adduction ROM. Also, outcomes achieved the minimum clinically important difference (MCID) [34,35]. Several possible mechanisms underlying the ROM improvements in overhead athletes have been described.

Wilk et al. [36] discussed that GIRD occurs due to the restriction of the posteroinferior capsule, the posterior band of the inferior glenohumeral ligament, and the stiffness of the posterior shoulder muscles [5]. The restriction of the tissues of the posterior part of the shoulder shifts the humeral head center of rotation to posterosuperior, generating an attenuation of the anterior part of the capsule and ligaments. The different stretching techniques in isolation or when included in the exercise therapy programs, and the manual therapy techniques, may improve the extensibility and mobility of the different tissues of the posterior part of the shoulder [20,26,27]. Consequently, the tension in the anterior part of the capsule and ligaments may decrease and restore the center of rotation of the humeral head. All these mechanical changes could explain the increment of the internal rotation and adduction ROM.

Concerning physical function, previous studies concluded that internal rotation ROM was positively directly correlated to physical function in overhead athletes [37]. Therefore, the outcomes achieved in internal rotation ROM may explain the improvements shown in physical function. The changes in physical function were statistically significant but did not achieve the MCID [35]. This fact could be related to the samples included in the studies; the participants were overhead athletes with high physical function levels, and could be less able to condition short-term effects after the intervention.

The shift of the humeral head center causes a functional narrowing of the subacromial space [38]. This functional narrowing of the subacromial space has been directly linked to glenohumeral instability in overhead athletes aged < 35 years old [39,40]. The results of the meta-analysis in this variable showed a statically significant increment after the intervention [27,29]. The change of the center of rotation of the humerus to the physiological position due to the stretching may explain the improvements achieved in this study.

Pain intensity, external rotation ROM and shoulder strength showed no statistically significant differences after the intervention. Pain is a complex subjective experience, including different dimensions of pain sensory-discriminative, affective-motivational and cognitive-evaluative factors [41]. The external rotation ROM showed no statistically significant changes. These results are in accordance with other authors that showed that external rotation ROM did not increase after a conservative intervention [42,43]. The ERG is a risk factor for the development of overuse injuries [5,44,45,46], so other authors have proposed that external rotation ROM should not increase after the intervention. It is thought that this aspect could decrease the degree of compression on the rotator cuff from the superior glenoid [47].

Regarding strength, the studies included in the meta-analysis showed no changes after conservative therapy. The studies included in the statistical analysis applied only passive therapies which may explain the lack of improvement. Our results are in agreement with previous studies that described no effects on strength after dry needling [48] or kinesio taping [49] applications. In the systematic review, two studies that applied therapeutic exercise showed statistically significant improvements [23,31]. Thus, the application of active therapies focused on strengthening seems to be necessary to achieve improvements in shoulder strength.

This systematic review and meta-analysis has some limitations. Our search strategy may have been limited by the omission of other databases, such as CINAHL, and we may have missed relevant articles. The heterogeneity found in the treatments applied in the studies, such as the type and the duration of the therapies applied, complicates the interpretation of our results. Methodological limitations include insufficient sample size, that could overestimate the results; and the lack of follow-up measurements of the studies, as none of the studies assessed the follow-up.

From a clinical point of view, the results of this study suggest that conservative therapy based on passive and active stretching, manual therapy, soft tissue mobilization, and exercise therapy, improves internal rotation and adduction ROM, physical function, and subacromial space in overhead athletes with GIRD. These results are an important factor in clinical practice and in practical sport as the internal rotation ROM deficit could be a risk factor to develop shoulder dysfunctions, as previous studies have demonstrated. Overhead athletes may avoid or decrease the risk of overuse injuries by using conservative therapies to improve internal rotation ROM.

## 5. Conclusions

Our findings show high-quality evidence that conservative therapy based on stretching, manual therapy and soft tissues mobilization are more effective than control, sham or other conservative therapies for improving internal rotation and adduction ROM and subacromial space; moderate-quality evidence for subacromial space; and low quality of evidence for physical function in overhead athletes with GIRD. This systematic review and meta-analysis demonstrate that these conservative therapies do not provide significant differences in pain relief and shoulder strength in overhead athletes with GIRD.

## Figures and Tables

**Table 1:** Characteristics of included studies.

Author (Year)	Participants				Intervention			Outcome (Tool)	Main Results	PEDro
	N (Sex Ratio)	Mean Age (SD)	Pathology	Sport	CG	EG	OG			
Maenhout et al., 2012 [25]	62 (40M/32F)	CG: 22.1 (2.2) EG: 21.4 (1.5)	Internal rotation deficits	Overhead sports: volleyball, handball, tennis, badminton	Control (*n* = 32)	Sleeper stretch (*n* = 30): 3 repetitions 30 s (6 weeks)		- ROM (digital inclinometer): IR, ER, Add/ - Subacromial space at 0°/45°/60°	↑ IR, Add ROM SS 0 and 45° in IG Vs CG	4/10
Bailey et al., 2017 [27]	60 (60M)	CG: 18.6 (2.1) EG: 18.8 (2.6)	Internal rotation deficits	Baseball	Supervised self-stretching (4 min) (*n* = 30)	Manual therapy (4 min) (instrumental) + Self-stretching (4 min) (*n* = 30)		- Shoulder ROM: (digital inclinometer) IR, ER and TR, Add - Humeral torsion (ultrasound)	↑ IR ROM in IG Vs CG ↑Add ROM in IG Vs CG	5/10
Chepeha et al. 2017 [28]	37 (20M/17F)	20.3 (1.4)	Internal rotation deficits	Overhead sport: volleyball, tennis swimming	Control (*n* = 17)	Stretching (*n* = 20): 8 week/once daily for 5 repetitions 2 min		- Shoulder ROM: (standard goniometer) IR, Add, GIRD - Pain (0–100) - Function (0–100)	↑IR Add Function	6/10
Lluch et al., 2018 [30]	31 (18M/13F)	28.7 (8.67)	Chronic shoulder pain	Overhead sport: handball, volleyball, tennis	Control (*n* = 10)	AP shoulder mobilization grade III (10 min, 3 sets of 3 min alternating 30 s rest) (*n* = 11)	Manual contact (*n* = 10)	- Pain (NPRS 0–10) - PPT (Fisher algometer) - ROM (goniometer) - Strength (hand-held dynamometer) - Disability (DASH)	No differences were found between groups	8/10
Lo et al., 2021 [29]	31 (31M)	20.36 (1.91)	Internal rotation deficits	Baseball	Control (*n* = 10)	Kinesiotaping (*n* = 11)	Sleeper stretch (*n* = 10)	- ROM (goniometer): IR, ER, Add - Strength (handheld dynamometer) - Subacromial space (ultrasound)	↑ IR ROM in EG and OG Vs CG	7/10
Gaharisia et al., 2021 [20]	40 (20M/20F)	25.9 (2.6)	Internal rotation deficits	Overhead sport: volleyball, baseball, tennis, waterpolo, squash, swimming		Novel stretch: passive glenohumeral rotation with clam shell bridging (*n* = 20)	Modified sleeper stretch (*n* = 20): (passive glenohumeral joint rotation) 4 weeks 30 s/30 s rest/3 rep	- Shoulder ROM: (digital inclinometer) Internal rotation ROM - Pain (NPRS)	↑Pain in EG Vs OG ↑ IR ROM in EG Vs OG	8/10
Ceballos et al., 2021 [32]	30 (30M)	22.39 (3.71)	Internal rotation deficits and shoulder pain	Handball	Sham dry needling (*n* = 15)	Dry needling teres major (*n* = 15)		- Pain (NPRS) - ROM (digital inclinometer): IR, ER, - Strength (handheld dynamometer) - Extensibility (digital inclinometer)	↑Pain in EG Vs CG ↑IR ROM and extensibility in EG Vs CG	9/10
Kamali et al., 2021 [26]	30 (30M)	EG:23.4 (4.79) OG:21.26 (2.98)	Internal rotation deficits	Volleyball		Stretching + scapular mobilization (*n* = 15)	Stretching (*n* = 15)	- ROM (digital photography): IR, ER, GIRD, ERG	No differences were found between groups	8/10
Moradi et al., 2020 [23]	60 (60M)	EG:23.9 (4.4) OG:23.4 (3.8)	Internal rotation deficits	Volleyball		supervised throwing exercise (*n* = 30) with a TheraBand 40 min/session, 3 sessions/week (8 weeks)	self-exercise program (*n* = 30): No strength exercises home self-exercise program for three 40-min sessions/week (8 weeks)	- ROM (goniometer): IR - EMG (surface) - Strength (isokinetic dynamometer) - Joint position sense	↑IR ROM, EMG activity of the anterior deltoid iddle deltoid posterior deltoid, infraspinatus and supraspinatus muscles, rotator cuff muscle strength ratio and joint position sense in EG Vs OG	6/10
Nelson et al., 2020 [24]	40 (No data)	EG: 23 (2.89) OG: 22 (3.05)	Internal rotation deficits	Overhead sports		Fascial manipulation (*n* = 20) (8–10 points/5–9 min 45 min session) 2 weeks	Shoulder posterior capsular ball release (*n* = 20): temporary obstruction of local blood flow + sleeper stretch (3 set of 30 s/1 min break)	- ROM: Internal rotation (universal goniometer)	No differences were found between groups	5/10
Sharma et al., 2021 [31]	80 (No data)	EG:21.3 (2.1) OG:21.8 (2.8)	Shoulder impingement	Overhead sports		Progressive resistance exercises + Manual therapy posteroanterior glides in thoracic spine + Glenohumeral posterior and inferior glide mobilizations (*n* = 40) (8 weeks)	Motor control exercise (*n* = 40)	- Isometric strength: handheld dynamometer: - UT, MTr, LT, SA, Supr, AD, LD	↑Isometric strength on UT: MTr, LT MA, SA, Supr, AD, LD, in EG Vs OG	6/10

CG, Control or sham group; EG, Experimental group; OG, Other conservative therapy group; M, Male; F, Female; ROM, range of movement; IR, internal rotation; ER, external rotation; Add, adduction; GIRD, glenohumeral internal rotation deficit; NPRS; numeric pain rating scale; PPT, pressure pain threshold; DASH; Disabilities of the arm, shoulder and hand, ERG, external rotation gain; EMG, electromyography; UT, upper trapezius; MTr, middle trapezius; LT, lower trapezius; SA, serratus anterior; Supr., supraspinatus; AD, anterior deltoid; LD, latissimus dorsi. ↑Statistically significant improvement.

## Data Availability

Not applicable.

## Appendix A. Detailed Search Strategy

**Table:**

P	I	C	O	S
(Athletes OR Sports OR Upper extremity OR young adult OR handball OR overhead) and (rotator cuff tendinopathy OR rotator cuff related pain OR shoulder impingement syndrome OR shoulder impingement OR non-specific shoulder pain OR joint instability OR Athletic injuries OR Shoulder injuries)	(Transcranial direct current stimulation) OR (physical therapy modalities) OR (isometric contraction) OR (exercise therapy) OR (exercise) OR (eccentric) OR (strength) OR (external rotation strength) OR (soft tissue) OR (mobilization) OR (physiotherapy)	(Control OR Sham OR Sham)	(Strength) OR (Range of motion) OR (Internal rotation) OR (external rotation) OR (GIRD) OR (pain) OR (myotonometry) OR (physical function)	Clinical trials Randomized clinical trials

#1 ((Athletes) OR (Sports *) OR (Upper extremity) OR (young adult) OR (handball) OR (overhead)). #2 ((rotator cuff tendinopathy) OR (rotator cuff related pain) OR (shoulder impingement syndrome) OR (shoulder impingement) OR (non-specific shoulder pain) OR (shoulder joint instability) OR (Shoulder injuries)). #3 ((physical therapy modalities) OR (isometric contraction) OR (exercise therapy) OR (exercise) OR (eccentric) OR (strength) OR (external rotation strength) OR (soft tissue) OR (mobilization) OR (physiotherapy) OR (Transcranial direct current stimulation)). #4((Control) OR (Sham) OR (Sham)). #5 ((Strength) OR (Range of motion) OR (Internal rotation) OR (external rotation) OR (GIRD) OR (pain) OR (myotonometry) OR (physical function)).

PUBMED

Formula: #1 AND #2 AND #3 AND #4 AND #5

Results: 175 Potential articles (PA)

SCOPUS

Formula: #1 AND #2 AND #3 AND #4 AND #5

Results: 535 PA

Web of Science

Formula: #1 AND #2 AND #3 AND #4

Results: 1575 PA

PEDro

Formula: #1

Results: 35 PA

COCHRANE

Formula: #1 AND #2 AND #3 AND #4

Results: 1791 PA

## Appendix B. GRADE Assessment of Conservative Therapy vs Control for Shoulder Dysfunctions in Overhead Athletes

**Table:**

			Certainty Assessment				N° of Patients		Effect		Certainty
N° of Studies	Study Design	Risk of Bias	Inconsistency	Indirectness	Imprecision	Other Considerations	Conservative Therapy	Control	Relative (95% IC)	Absolute (95% IC)	
			Internal Rotation ROM (Increasing values indicate improvement)								
8	RCT	Not serious	Not serious	Not serious	Not serious	none	171	169	-	SMD 1.09 higher (0.52 higher to 1.66)	⨁⨁⨁⨁ HIGH
			External rotation ROM (Increasing values indicate improvement)								
8	RCT	Not serious	Not serious	Not serious	Not serious	none	130	132	-	SMD 1.92 higher (6.11 higher to 2.27 lower)	⨁⨁⨁⨁ HIGH
			Adduction ROM (Increasing values indicate improvement)								
4	RCT	Not serious	Not serious	Not serious	Not serious	none	97	102	-	SMD 4.29 higher (0.25 higher to 8.33 higher)	⨁⨁⨁⨁ HIGH
			Internal rotation strength (Increasing values indicate improvement)								
3	RCT	Not serious	Not serious	Not serious	Not serious	none	56	55	-	SMD 0.24 lower (1.53 lower to 1.05 higher)	⨁⨁⨁⨁ HIGH
			External rotation strength (Increasing values indicate improvement)								
3	RCT	Not serious	Not serious	Not serious	Not serious	none	56	55	-	SMD 0.05 higher (−0.32 lower, −0.43 higher)	⨁⨁⨁⨁ HIGH
			Pain intensity (Decreasing values indicate improvement)								
4	RCT	Not serious	Not serious	Not serious	serious	none	75	72	-	SMD = 0.02 higher (1.45 lower, −1.48 higher)	⨁⨁⨁◯ MODERATE
			Physical function (Decreasing values indicate improvement)								
2	RCT	Not serious	Not serious	Not serious	serious	none	56	55	-	SMD −0.75 higher (−1.22 lower, −0.28 higher)	⨁⨁◯◯ LOW
			Subacromial space (Increasing values indicate improvement)								
2	RCT	Not serious	Not serious	Not serious	serious	none	51	52	-	SMD 0.62 higher (0.07 lower, 1.18 higher)	⨁⨁⨁◯ MODERATE

IC, Confidence Interval; RCT, randomized clinical trial; SMD, standard mean difference. ⊕⊕⊕⊕: High certainty, ⊕⊕⊕◯: Moderate certainty, ⊕⊕◯◯: Low certainty.
